# Differential introgression and the maintenance of species boundaries in an advanced generation avian hybrid zone

**DOI:** 10.1186/s12862-016-0635-y

**Published:** 2016-03-22

**Authors:** Jennifer Walsh, W. Gregory Shriver, Brian J. Olsen, Adrienne I. Kovach

**Affiliations:** Department of Natural Resources and the Environment, University of New Hampshire, Durham, NH USA; Department of Entomology and Wildlife Ecology, University of Delaware, Newark, DE USA; School of Biology and Ecology, University of Maine, Orono, ME USA; Cornell Lab of Ornithology, Cornell University, Ithaca, NY USA

**Keywords:** *Ammodramus caudacutus*, *Ammodramus nelsoni*, Introgression, Genomic clines, Geographic clines, Hybrid zones, Haldane’s rule

## Abstract

**Background:**

Evolutionary processes, including selection and differential fitness, shape the introgression of genetic material across a hybrid zone, resulting in the exchange of some genes but not others. Differential introgression of molecular or phenotypic markers can thus provide insight into factors contributing to reproductive isolation. We characterized patterns of genetic variation across a hybrid zone between two tidal marsh birds, Saltmarsh (*Ammodramus caudacutus*) and Nelson’s (*A. nelsoni*) sparrows (*n* = 286), and compared patterns of introgression among multiple genetic markers and phenotypic traits.

**Results:**

Geographic and genomic cline analyses revealed variable patterns of introgression among marker types. Most markers exhibited gradual clines and indicated that introgression exceeds the spatial extent of the previously documented hybrid zone. We found steeper clines, indicating strong selection for loci associated with traits related to tidal marsh adaptations, including for a marker linked to a gene region associated with metabolic functions, including an osmotic regulatory pathway, as well as for a marker related to melanin-based pigmentation, supporting an adaptive role of darker plumage (salt marsh melanism) in tidal marshes. Narrow clines at mitochondrial and sex-linked markers also offer support for Haldane’s rule. We detected patterns of asymmetrical introgression toward *A. caudacutus*, which may be driven by differences in mating strategy or differences in population density between the two species.

**Conclusions:**

Our findings offer insight into the dynamics of a hybrid zone traversing a unique environmental gradient and provide evidence for a role of ecological divergence in the maintenance of pure species boundaries despite ongoing gene flow.

**Electronic supplementary material:**

The online version of this article (doi:10.1186/s12862-016-0635-y) contains supplementary material, which is available to authorized users.

## Background

Hybrid zones are excellent model systems for evolutionary studies as they provide a diversity of recombinant genotypes through generations of mutation, recombination, and gene flow [1, 2]. Growing empirical evidence indicates that natural hybrid zones occur across a range of taxonomic groups at rates greater than previously estimated [3] and that hybridization and introgression are important forces that can shape the evolutionary trajectory of a species [4–6]. Studies of hybridizing taxa that maintain genetic distinction with ongoing gene flow provide insight into the speciation process [6, 7] and offer a direct measure of reproductive isolation. Because hybrid zone studies allow for the quantification of differential patterns of introgression of foreign alleles, hybrid zones provide the opportunity to identify the genetic and phenotypic traits influencing species divergence [8].

Hybrid zones are thought to be semi-permeable boundaries between genomes as differential fitness of hybrids can result in reduced introgression of those regions important in maintaining reproductive isolation, while introgression of regions free of selection is permitted [1, 9, 10]. Loci with no influence on hybrid fitness should display uninhibited movement across a hybrid zone, whereas regions underlying local adaptation or that are responsible for genetic incompatibilities remain differentiated, often in the presence of gene flow [1, 8, 11]. Rates of introgression have been found to vary among genetic and phenotypic markers across a number of natural hybrid zones [12–14]. These observations have been linked to numerous demographic and selective processes, including genetic incompatibilities [15], ecological divergence [16], differential fitness [17], and variations in mate preference and behavior [11].

Sampling a diversity of genetic and phenotypic markers provides an unbiased view of introgression and genetic structure across a hybrid zone [18, 19]. Understanding these patterns can offer valuable insight into the mechanisms responsible for restricting gene flow across species’ boundaries [11, 20–22], as differential introgression may be indicative of ecological or evolutionary dynamics in the focal gene regions [13, 23]. For example, neutral microsatellite markers should diffuse freely across the hybrid zone, resulting in widespread movement of alleles. Conversely, diagnostic markers (i.e. markers that are fixed or highly differentiated between two parental species) are predicted to be under divergent selection, exhibiting reduced introgression [19], as the elevated divergence typically associated with diagnostic markers suggests association with genomic regions under selection [24]. Differential introgression of sex-linked and mitochondrial markers relative to autosomal loci is often attributed to Haldane’s rule, which predicts greater fitness reductions in hybrids of the heterogametic sex [25]. This pattern has been observed in a number of avian [26–28] and mammalian systems [12].

Morphological traits also provide insight into extrinsic selection and demographic events shaping a hybrid zone [29]. Bimodal distribution of phenotypes, or an abrupt clinal transition, can be indicative of high dispersal, differential selection, hybrid zone movement [13, 30], or assortative mating [29, 31]. Assessing introgression of secondary sexual characteristics (e.g., plumage) can also aid in identifying patterns of asymmetrical introgression [11]. Divergence in plumage characteristics can be particularly important in driving pre-zygotic isolation in birds [32], as these traits play an important role in mate selection, providing a range of important cues to females including individual and territory quality [33, 34] and offspring attentiveness [35].

Here we investigated patterns of introgression in an avian hybrid zone between two recently diverged marsh endemics, the Saltmarsh (*Ammodramus caudacutus*) and Nelson’s (*A. nelsoni*) sparrow (~600,000 years; [36]). In the USA and Maritime Canada, the two species are restricted to a linear ribbon of tidal-marsh habitat along the Atlantic seaboard with a subspecies of *caudacutus* (*A.c. caudacutus*) predominantly inhabiting coastal salt marshes from southern Maine to New Jersey and a subspecies of *nelsoni* (*A.n. subvirgatus*) predominantly inhabiting brackish and tidal marshes from the Canadian Maritimes to northern Massachusetts [37, 38]. Current knowledge suggests that the two taxa (hereafter *caudacutus* and *nelsoni*, respectively) overlap and hybridize in tidal marshes along a 210 km stretch of the New England coast between the Weskeag River estuary in South Thomaston, Maine and Plum Island in Newburyport, Massachusetts [39–41].

Recent work in the *caudacutus-nelsoni* hybrid zone indicates extensive introgression with a high proportion of backcrossed sparrows in sympatric populations [42]. Despite high rates of admixture, very few individuals are recent generation (F1/F2) hybrids (3 %; [42]), indicative of an advanced generation hybrid zone characterized by high rates of recombination [43, 44]. Accordingly, there is no intermediate hybrid phenotype, and complex patterns of morphological variation preclude discrimination of pure and admixed sparrows from morphology alone [42]. While backcrossing is extensive between *caudacutus* and *nelsoni*, variation in habitat affinity, morphology, and behavior suggest a role for isolating mechanisms in this system. Abrupt environmental gradients across the marine-terrestrial ecotone within tidal marshes present adaptive challenges to terrestrial vertebrates (e.g. tidal inundation and osmoregulatory demands [45, 46]). *A. caudacutus* is a narrow niche specialist, reliant exclusively on salt marshes in both its breeding and wintering habitat; it has been associated with salt marshes over a longer evolutionary time frame [47] compared to *A. nelsoni*, which, in allopatry, uses a broader range of habitats including brackish and fresh water marshes and hay fields. In the hybrid zone, a mosaic of fine-scale habitat types – coastal, bay, and upriver, tidal marshes – occurs, and the spatial structuring of pure and hybrid individuals follows a patchy distribution consistent with these local habitat differences [48]. Due to these differences in niche specificity, there may be stronger selection for adaptive traits in pure *caudacutus* individuals, driving ecological divergence. Tidal marsh adaptations may also influence morphology and plumage coloration in pure *caudacutus* and *nelsoni* [42, 49, 50] with potential reinforcement of these traits through sexual selection. Numerous behavioral differences between *caudacutus* and *nelsoni* males, including differences in flight displays, song, aggressiveness, and mating strategy [37, 38, 51] further have the potential to shape asymmetries in mate selection within the hybrid zone.

The aim of this study was to characterize the genetic structure, including patterns of differential introgression and selection, across the *caudacutus-nelsoni* hybrid zone and to test the hypothesis that adaptive traits are important in maintaining pure species boundaries despite ongoing gene flow. We conducted extensive, systematic sampling across the full extent of the *caudacutus-nelsoni* hybrid zone, coupled with population genetic analyses and geographic and non-geographic cline analyses to characterize genetic variation, quantify introgression across genetic and morphological markers, and identify the width and center of the hybrid zone. We used plumage features and a diversity of genetic markers, including anonymous (putatively neutral) microsatellites, diagnostic (species-specific and potentially under selection; [52]) microsatellites, mitochondrial, and sex-linked markers, to compare introgression patterns across potentially variable selective processes. The diagnostic markers used in this study were identified from a genome-wide comparison of microsatellite loci between *caudacutus* and *nelsoni* [52] and were selected because they showed elevated divergence between allopatric *caudacutus* and *nelsoni* individuals (*F*_*ST*_ = 0.4667) compared to neutral, anonymous microsatellites (*F*_*ST*_ = 0.15). Several of the loci are linked to genes of known function, indicative of divergent selection for functional traits that differ between the species [52]. The potential influence of selection and introgression patterns for these markers across the geographic extent of the hybrid zone are of yet unknown. We predicted that the gene-associated diagnostic markers would show reduced introgression and more abrupt clines compared to the neutral microsatellite markers. We also predicted selection would occur for sex-linked and mitochondrial markers in accordance with Haldane’s rule. In birds, females are the heterogametic sex (ZW), and thus Haldane’s rule predicts reduced introgression of both sex-linked markers and mitochondrial markers (due to maternal inheritance) compared to autosomal markers. Lastly, we predicted strong selection for features related to plumage darkness, as increased melanin is thought to be an adaptation to tidal marshes (salt marsh melanism; [49, 53, 54]), which we hypothesized to be under stronger selection in *caudacutus*.

## Results

**Fig. 1:**
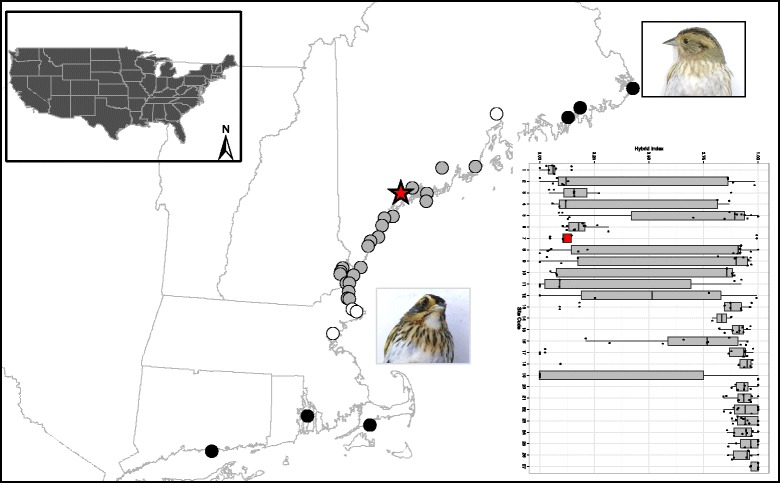
The location of 32 marshes along the northeastern coast of the United States where *A. caudacutus* and *A. nelsoni* individuals were sampled. Black circles represent allopatric populations from which putatively pure individuals were used for calculating a hybrid index. White circles represent marshes that are outside of the currently hypothesized overlap zone, yet were treated as sympatric populations due to their close proximity to the hybrid zone and evidence of introgressed individuals [50]. Gray circles represent marshes within the hybrid zone and the red star represents the approximate center of the zone, based on geographic cline estimates. The boxplot represents the distribution of hybrid index values for each of the sympatric marshes sampled; the center of the hybrid zone is colored in red. To demonstrate phenotypic differences between the parental species, representative photographs are shown for allopatric *nelsoni* (*top*) and *caudacutus* (*bottom*). Hybrids do not exhibit a clear intermediate phenotype, but rather display complex combinations of phenotypic traits that do not vary predictably by genotypic class ([42]; see text)

We characterized genotypic data at 24 microsatellite loci and DNA sequences from two mitochondrial, 2 Z-linked and one autosomal gene from 286 sparrows from 32 marshes across the *caudacutus-nelsoni* hybrid zone and surrounding allopatric populations (Fig. 1). We obtained morphological data (plumage, bill, and structural measurements) from 254 of these individuals. Microsatellite loci were highly polymorphic with allelic richness ranging from 4 to 33 alleles per locus (mean = 12.7). Allelic richness was greater in pure *caudacutus* populations (mean = 8.5 alleles per locus, range = 2 – 21) than in pure *nelsoni* populations (mean = 7.3 alleles per locus, range = 1 – 14). Mean observed heterozygosities ranged from 0.531 to 0.704 (Table 1), with heterozygosity generally increasing from North to South. Six of the 24 microsatellite markers (*Ammo*008, *Ammo*012, *Ammo*015, *Ammo*016, *Ammo*030, and *Ammo*036) were candidates for positive selection, likely a result of their association with coding regions (Additional file 1: Figure S1). All other microsatellite markers were within neutral expectations. We detected significant deviations (Bonferroni adjustment; α = 0.05, *P* = 0.001) from Hardy-Weinberg in 7 out of 32 (22 %) marshes (Table 1). We did not observe significant linkage disequilibrium in any of our populations.

**Table 1:** Sampling locations and descriptive statistics for *A. caudacutus* and *A. nelsoni*. Table includes marsh names, distance along the geographic transect, sampling coordinates, sample size, observed (HO) and expected (HE) heterozygosity, *F*_*IS*_, average Q values, hybrid index, and interspecific heterozygosity for each marsh based on analyses in structure and *introgress*.

Sampling location	Distance from locality 1 (km)	Latitude	Longitude	*n*	HO	HE	FIS	Q Value (average)	Hybrid Index (average)	Interspecific Heterozygosity (Average)
Lubec, ME	0	44.822	−66.991	9	0.537	0.555	0.032	0.021	NA	NA
Columbia Falls, ME	61	44.644	−67.719	10	0.583	0.563	−0.037	0.003	NA	NA
Narraguagus River - Millbridge, ME	78	44.551	−68.891	9	0.542	0.551	0.018	0.002	NA	NA
Mendell Marsh - Penobscot, ME	155	44.591	−68.859	9	0.583	0.555	−0.050	0.004	0.06	0.38
Weskeag Marsh - South Thomaston, ME	216	44.077	−69.142	9	0.592	0.760	0.221*	0.430	0.44	0.31
Sheepscot River - Newcastle, ME	252	44.065	−69.597	7	0.643	0.697	0.077	0.185	0.24	0.42
Morse Cove - Arrowsic, ME	287	43.816	−69.795	5	0.617	0.799	0.228	0.390	0.39	0.36
Popham Beach - Phippsburg, ME	292	43.739	−69.806	15	0.675	0.761	0.113*	0.714	0.69	0.28
Maquoit Bay - Brunswick, ME	313	43.867	−69.988	10	0.613	0.618	0.008	0.083	0.18	0.48
Cousins River - Yarmouth, ME	328	43.811	−70.156	5	0.614	0.714	0.100	0.201	0.28	0.37
Spurwink River - Cape Elizabeth, ME	358	43.588	−70.246	16	0.667	0.779	0.143*	0.632	0.61	0.22
Scarborough Marsh - Scarborough, ME	367	43.575	−70.372	14	0.627	0.773	0.189*	0.645	0.67	0.27
Saco River - Saco, ME	376	43.492	−70.391	7	0.619	0.784	0.211*	0.566	0.53	0.29
Marshall Point - Arundel, ME	388	43.381	−70.433	6	0.583	0.766	0.239	0.334	0.33	0.28
Little River - Wells, ME	398	43.344	−70.538	4	0.594	0.788	0.246	0.540	0.51	0.33
Eldridge Marsh - Wells, ME	404	43.292	−70.572	9	0.652	0.783	0.166	0.760	0.74	0.25
Seapoint - Kittery Point, ME	432	43.087	−70.664	9	0.648	0.691	0.063	0.984	0.90	0.21
Lubberland Creek - Newmarket, NH	452	43.073	−70.903	10	0.704	0.772	0.088	0.747	0.70	0.38
Chapman’s Landing - Stratham, NH	456	43.041	−70.924	10	0.583	0.745	0.217*	0.796	0.74	0.21
Squamscott River - Exeter, NH	458	43.017	−70.935	6	0.653	0.723	0.095	0.832	0.81	0.21
Awcomin Marsh - Rye, NH	473	43.006	−70.752	7	0.531	0.788	0.326*	0.429	0.33	0.23
Drakeside Marsh - Hampton, NH	485	42.931	−70.852	7	0.702	0.678	−0.036	0.995	0.93	0.19
Hampton Beach - Hampton, NH	489	42.926	−70.806	9	0.694	0.681	−0.020	0.992	0.93	0.22
Salisbury Marsh - Salisbury, MA	498	42.844	−70.822	10	0.633	0.691	0.084	0.991	0.94	0.18
Pine Island - Newburyport, MA	505	42.775	−70.827	13	0.660	0.664	0.005	0.996	0.94	0.20
Plum Island - Newburyport, MA	507	42.774	−70.809	9	0.694	0.702	0.011	0.989	0.93	0.19
Castle Hill - Ipswich, MA	512	42.679	−70.773	7	0.702	0.675	−0.040	0.998	0.95	0.17
Farm Creek Marshes - Gloucester, MA	526	42.658	−70.708	10	0.639	0.716	0.107	0.993	0.93	0.16
Rever, MA	565	42.436	−71.011	5	0.617	0.688	0.104	0.997	0.98	0.14
Monomoy Island - Chatham, MA	688	41.603	−69.987	11	0.598	0.646	0.074	0.998	NA	NA
Prudence Island - Jamestown, RI	800	41.647	−71.343	9	0.606	0.639	0.053	0.998	NA	NA
Hammonasset Beach - Madison, CT	910	41.263	−72.551	10	0.642	0.705	0.089	0.997	NA	NA

*F*_*IS*_ significance is indicated by an asterisk

### Genetic structure of the *caudacutus-nelsoni* hybrid zone

**Fig. 2:**
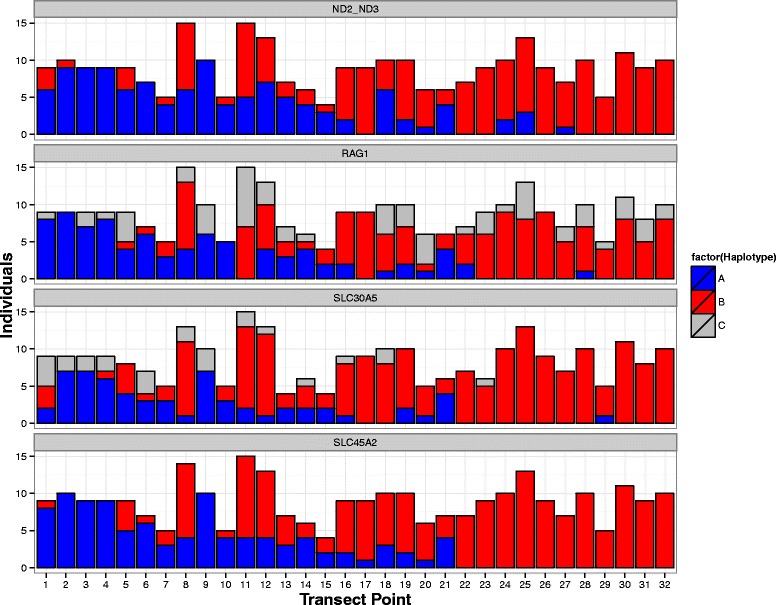
Bar plot showing the distribution of haplotypes by transect point (*nelsoni* haplotypes are blue, *caudacutus* haplotypes are in red, and hybrid haplotypes are in gray). Each panel represents a marker, from top to bottom: ND3/ND2 (mitochondrial), RAG1 (nuclear), SLC45A2 (z-linked), and SLC30A5 (z-linked). The hybrid zone is located between sampling points 4 and 27

**Fig. 3:**
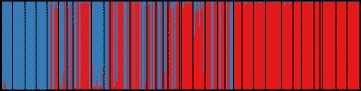
Population clusters identified by structure for 286 individuals genotyped at 24 microsatellite loci. Bar plot shows individual membership to two genetic clusters. Blue represents *nelsoni* genotypes and red represent *caudacutus* genotypes

Haplotype distributions among sampling locations varied by marker, with the least mixing observed in ND2, ND3, and SLC45A2 (Fig. 2); because ND2 and ND3 were identical for all individuals, they are combined for subsequent descriptions. We detected *caudacutus* haplotypes in our putatively allopatric *nelsoni* populations for four out of five genes: 4 individuals (10 %) for ND2/ND3, 1 individual (2 %) for SLC45A2, and 4 individuals (10 %) for SLC30A5. We detected fewer instances of *nelsoni* haplotypes in putatively pure *caudacutus* populations, with only 1 individual (2 %) for ND2/ND3, SLC30A5, and RAG-1. We assigned hybrid haplotypes for the two markers with RFLP banding patterns (RAG-1 and SLC30A5). In allopatric populations, we identified 4 putatively pure *nelsoni* (11 %) and 9 putatively pure *caudacutus* (25 %) with mixed haplotypes for RAG-1. For SLC30A5, we found hybrid haplotypes in the pure *nelsoni* populations (27 %) but no hybrid haplotypes in the pure *caudacutus* populations. In sympatric marshes, the percentages of *nelsoni* and *caudacutus* haplotypes were as follows: 41 % *nelsoni* and 59 % *caudacutus* for ND2/ND3 and 31 % *nelsoni* and 69 % *caudacutus* for SLC45A2. For SLC30A5 and RAG-1, the percentages of *nelsoni*, *caudacutus*, and hybrid haplotypes were 20, 71, 8 and 26, 50, 24 %, respectively (Fig. 2).

structure assigned individuals to one of two genetic clusters (Fig. 3) based on Δ*K* (Additional file 2: Figure S2), which corresponded to *caudacutus* and *nelsoni* populations. Consistent with previous findings, we found few intermediate individuals (F1 hybrids) and pure and backcrossed individuals appeared to be patchily distributed across sympatric populations (Fig. 3). Individuals sampled from allopatric *nelsoni* populations had a low probability (mean Q value ± SD = 0.007 ± 0.01) of being assigned to the *caudacutus* cluster, while individuals sampled from allopatric *caudacutus* populations had a high probability of being assigned to the *caudacutus* cluster (mean Q ± SD = 0.995 ± 0.006). Sympatric populations had intermediate Q values and hybrid indices (mean Q ± SD = 0.667 ± 0.450, Range = 0 – 1 and mean HI ± SD = 0.66 ± 0.38, Range = 0 – 1); however pure *caudacutus* and pure *nelsoni* individuals inhabiting the same marshes largely drove this pattern (Fig. 3). Twelve individuals (4 %) had Q values ranging from 0.1 to 0.9 (recent generation hybrids); these 12 individuals were dispersed across the sampled marshes (i.e., there were no marshes with a disproportionately high number of recent generation hybrids). There were 94 individuals (42 %) with a hybrid index ranging from 0.1 to 0.9 (indicating they were not pure parental genotypes). Mean site-specific interspecific heterozygosity ranged from 0 to 0.76 (mean ± SD = 0.26 ± 0.12), with the greatest interspecific heterozygosities found on sites near the center of the hybrid zone (Table 1). We observed significant genetic differentiation among sampled marshes (*F*_*ST*_), with values ranging from 0 to 0.375 (θ = 0.1; Additional file 3: Figure S3). The largest *F*_*ST*_ values were generally observed between the allopatric *nelsoni* populations and all other marshes. We also detected significant *F*_*ST*_ values between sympatric marshes that were predominantly composed of *nelsoni* individuals (Maquoit Bay, Cousins River, and Rye Beach) compared to all other marshes.

### Genomic and geographic analyses of introgression

**Fig. 4:**
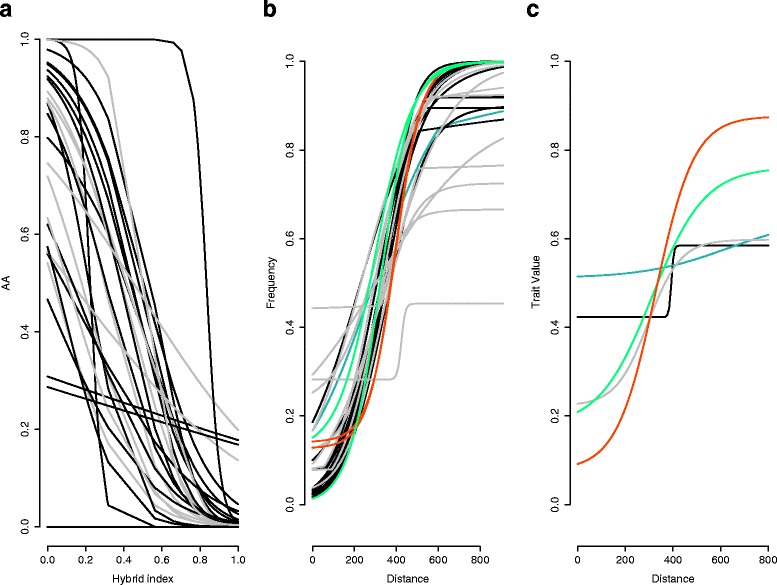
Plots showing patterns of genomic and geographic introgression across 32 *A. caudacutus* and *A. nelsoni* populations (*n* = 286). **a** Genomic clines calculated in *introgress* plotted as the observed frequency of *A.* caudacutus homozygote genotypes (1.0) against the hybrid index (calculated as the proportion of *A. caudacutus* alleles across all loci). Black lines show markers that deviate significantly from neutral introgression and gray lines show markers that do not deviate from neutral patterns of introgression. **b** Geographic clines calculated for 29 markers plotted as the frequency of *caudacutus* alleles across an 800 km sampling transect. Neutral markers are in gray, diagnostic markers in black, mitochondrial markers in red, z-linked markers in green, and an autosomal marker in blue. **c** Geographic clines calculated for five morphological traits. Weight is in black, wing chord in blue, bill length in gray, plumage amount in green, and plumage definition in red

**Fig. 5:**
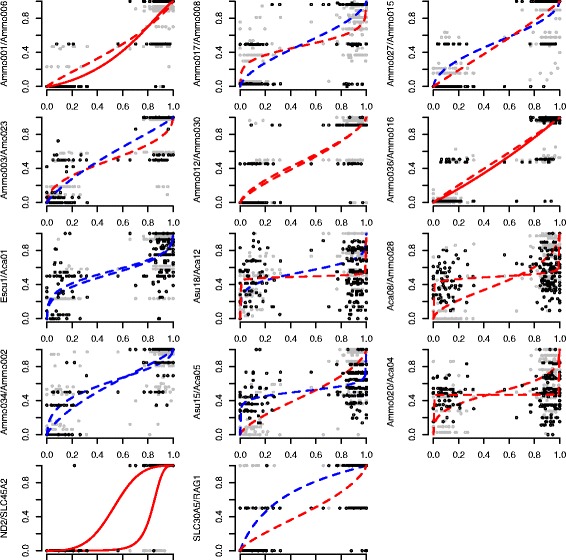
Analysis of introgression patterns comparing hybrid index for a focal locus (*y axis*) versus hybrid index for a multilocus expectation (*x axis*). Clines were compared using the logit-logistic model of Fitzpatrick [99]. Two loci are presented in each plot; black and gray points represent the raw data points for each marker. Line color indicates whether markers are shifted toward *nelsoni* (*blue*) or *caudacutus* (*red*) based on estimates of *u*. Line type indicates whether markers show gradual (*dotted*) or abrupt slopes (*solid*) based on estimates of *v*

Genomic clines revealed that introgression patterns were variable among markers (Additional file 4: Figure S4). Sixty-six percent (19) of the 29 markers showed deviations from patterns of neutral introgression (meaning they either exhibited more gradual or more abrupt patterns compared to neutral expectation; Fig. 4, Additional file 5: Figure S5). Clines were steeper than neutral expectation for 12 of these markers, including six of the diagnostic microsatellite loci (*Ammo* markers 001, 003, 006, 008, 027, 036), three of the anonymous microsatellites (*Escμ*1, *Asμ*15, *Aca*08), two mitochondrial markers (ND2/ND3), and SLC30A5. Six neutral microsatellite markers and RAG-1 displayed more gradual clines than neutral expectation. Comparison of individual loci to multilocus expectation using the logit-logistic model revealed variations in cline slope and position among the 29 genetic markers (Fig. 5, Table 2). We detected overall patterns of asymmetrical introgression with 66 % (19) of the markers shifted toward *caudacutus* and 34 % (10 loci) shifted toward *nelsoni*. Five markers (*Ammo*006, *Ammo*036, ND2, ND3, and SLC45A2) displayed stronger selection (more abrupt slopes; Table 2); all five of these markers exhibited asymmetrical introgression toward *caudacutus*. Twenty-four markers displayed more gradual slopes (weaker selection) than multilocus expectation.

**Table 2:** Summary of *v* and *u* estimates from concordance tests. These parameters were estimated based on the comparison of each focal locus to a multilocus expectation using a logit-logistic model. Parameter estimates are presented for 24 microsatellite loci, 2 mitochondrial genes, 2 z-linked genes, and 1 autosomal locus. Perfect concordance between a focal locus and the multilocus expectation is a diagonal line (*u* = 0 and *v* =1)

Locus	Asymmetry (*u*)		Slope (ν)	
	*nelsoni*	*caudacutus*	Gradual	Abrupt
Ammo001		+0.359	−0.124	
Ammo006		+0.822		+0.217
Ammo017		+0.077	−0.670	
Ammo008	−0.007		−0.306	
Ammo027	−0.08		−0.366	
Ammo015		+0.098	−0.035	
Ammo003		+0.009	−0.451	
Ammo023	−0.274		−0.117	
Ammo012		+0.146	−0.221	
Ammo030		+0.026	−0.275	
Ammo036		+0.234		+0.013
Ammo016		+0.028	−0.037	
Escu1	−0.083		−0.564	
Aca01	−0.220		−0.590	
Asu18	−0.197		−0.706	
Aca12		+0.028	−0.958	
Aca08		+0.429	−0.379	
Ammo028		+0.028	−0.915	
Ammo034	−0.485		−0.461	
Ammo002	−0.332		−0.175	
Asu15		+0.198	−0.279	
Aca05	−0.046		−0.832	
Ammo020		+0.019	−0.596	
Aca04		+0.125	−0.998	
ND2		+4.892		+2.014
ND3		+4.892		+2.014
SLC45A2		+0.259		+1.414
SLC30A5	−1.104		−0.073	
RAG1		+0.264	−0.236	

Geographic cline analyses revealed variation in estimates for cline width (mean = 392 km, range = 248 – 969) but more consistent estimates for cline center (mean = 330 km, range = 229 – 421; Table 3) across marker types. Based on these estimates, cline center was consistently predicted to be around sampling location 10 (Cousins River – Yarmouth, Maine). Estimates for cline width were the smallest for mitochondrial (264 km) and z-linked markers (299 and 358 km for SLC45A2 and SLC30A5, respectively), followed by diagnostic microsatellite markers (mean ± SD = 390 ± 83). Estimates for cline width were largest and most variable for the neutral microsatellite markers (426 ± 235). Similar to the cline estimates for the genetic markers, cline width was variable for the morphological traits (mean = 231 km, range = 17 – 450) and more consistent for cline center (338 km, range = 284 – 392; Table 4).

**Table 3:** Parameter estimates for the best fitting clines for 29 markers, including (in order from top to bottom): 12 diagnostic microsatellites, 12 anonymous microsatellites, 2 mitochondrial markers, 2 z-linked markers, and 1 autosomal marker. Geographic clines were fit using the R package *HZAR*. For each locus, we present the top model, estimates for cline width (*w*), cline center (c), *pMin/pMax (*allele frequencies at the end of the cline), estimates for the shape of the left, right, and mirrored tails, and the AICc

Locus	Best model	ω	c	ρmin	ρmax	deltaL	tauL	deltaR	tauR	deltaM	tauM	AICc - 15-model best fit
Ammo001	Pmin/Pmax fixed, no tails	401.26 (313.01 – 532.13)	325.42 (288.98 – 356.22)	0 (fixed)	1 (fixed)	NA	NA	NA	NA	NA	NA	59.40733
Ammo006	Pmin/Pmanx fixed, left tail	248.12 (142.16 – 420.99)	349.82 (294.33 – 393.54)	0 (fixed)	1 (fixed)	0.85	0.5	NA	NA	NA	NA	51.26572
Ammo017	Pmin/Pmax fixed, no tails	619.85 (469.9 – 871.3)	229.08 (158.21 – 279.31)	0 (fixed)	1 (fixed)	NA	NA	NA	NA	NA	NA	38.49596
Ammo008	Pmin/Pmax fixed, mirror tails	430.16 (341.34 – 598.06)	332.66 (292.59 – 361.66)	0 (fixed)	1 (fixed)	NA	NA	NA	NA	271.5	0.0	48.94506
Ammo027	Pmin/Pmax fixed, right tail	377.28 (292.21 – 511.31)	350.59 (318.50 – 378.73)	0 (fixed)	1 (fixed)	NA	NA	156.78	0.07	NA	NA	44.69117
Ammo015	Pmin/Pmax fixed, no tails	336.68 (263.38 – 441.98)	333.40 (301.87 – 360.36)	0 (fixed)	1 (fixed)	NA	NA	NA	NA	NA	NA	50.7184
Ammo003	Pmin/Pmax observed, no tails	393.52 (290.96 – 557.14)	349.81 (312.31 – 382.95)	0	0.9	NA	NA	NA	NA	NA	NA	29.95087
Ammo023	Pmin/Pmax fixed, right tail	328.79 (260.80 – 438.51)	336.03 (306.35 – 362.53)	0 (fixed)	1 (fixed)	NA	NA	175.86	0.00	NA	NA	52.34138
Ammo012	Pmin/Pmax fixed, no tails	377.14 (294.25 – 498.37)	331.05 (296.75 – 360.29)	0 (fixed)	1 (fixed)	NA	NA	NA	NA	NA	NA	38.59027
Ammo030	Pmin/Pmax fixed, mirror tails	414.72 (301.59 – 545.14)	303.25 (266.38 – 339.25)	0 (fixed)	1 (fixed)	NA	NA	NA	NA	240.3	0.0	45.33692
Ammo036	Pmin/Pmax fixed, no tails	390.91 (304.64 – 517.59)	329.39 (294.13 – 359.47)	0 (fixed)	1 (fixed)	NA	NA	NA	NA	NA	NA	42.29739
Ammo016	Pmin/Pmax fixed, no tails	355.08 (277.59 – 467.45)	330.41 (297.40 – 358.50)	0 (fixed)	1 (fixed)	NA	NA	NA	NA	NA	NA	51.76902
Escu1	Pmin/Pmax estimated, no tails	324.79 (164.62 – 564.91)	305.76 (236.92 – 375.68)	0.02	0.71	NA	NA	NA	NA	NA	NA	45.11181
Aca01	Pmin/Pmax fixed, no tails	573.52 (438.09 – 794.12)	230.56 (165.18 – 278.01)	0 (fixed)	1 (fixed)	NA	NA	NA	NA	NA	NA	27.04802
Asu18	Pmin/Pmax estimated, no tails	278.57 (64.34 – 676.36)	279.66 (166.01 – 368.15)	0.11	0.67	NA	NA	NA	NA	NA	NA	40.47708
Aca12	Pmin/Pmax observed, no tails	969.57 (607.18 – 969.99)	313.39 (229.74 – 395.79)	0.125	0.889	NA	NA	NA	NA	NA	NA	22.28862
Aca08	Pmin/Pmax fixed, no tails	613.14 (464.93 – 853.43)	350.56 (303.09 – 391.63)	0 (fixed)	1 (fixed)	NA	NA	NA	NA	NA	NA	36.1816
Ammo028	Null Model	NA	NA	NA	NA	NA	NA	NA	NA	NA	NA	30.08239
Ammo034	Pmin/Pmax fixed, mirror tails	405.81 (309.38 – 547.61)	295.14 (254.16 – 328.33)	0 (fixed)	1 (fixed)	NA	NA	NA	NA	239.7	0.086	35.99985
Ammo002	Pmin/Pmax observed, no tails	327.93 (239.38 – 456.58)	313.40 (272.38 – 346.71)	0.109	1	NA	NA	NA	NA	NA	NA	41.30775
Asu15	Pmin/Pmax fixed, mirror tails	394.21 (295.34 – 556.81)	330.15 (290.51 – 361.28)	0 (fixed)	1 (fixed)	NA	NA	NA	NA	237.2	0.013	37.69459
Aca05	Pmin/Pmax observed, mirror tails	341.90 (53.34 – 921.18)	421.24 (357.34 – 469.42)	0.278	0.929	NA	NA	NA	NA	87.45	0.002	18.60483
Ammo020	Pmin/Pmax observed, no tails	418.56 (291.17 – 625.28)	333.47 (280.87 – 374.64)	0.222	1	NA	NA	NA	NA	NA	NA	41.08943
Aca04	Pmin/Pmax estimated, no tails	37.73 (0 – 434.56)	419.01 (337.95 – 483.85)	0.283	0.454	NA	NA	NA	NA	NA	NA	24.34919
ND2	Pmin/Pmax estimated, no tails	264.11 (172.17 – 373.17)	391.96 (356.44 – 430.66)	0.129	0.999	NA	NA	NA	NA	NA	NA	80.13945
ND3	Pmin/Pmax estimated, no tails	264.11 (172.17 – 373.17)	391.96 (356.44 – 430.66)	0.129	0.999	NA	NA	NA	NA	NA	NA	80.13945
SLC45A2	Pmin/Pmax fixed, no tails	299.01 (233.91 – 390.53)	322.41 (292.12 – 348.18)	0 (fixed)	1 (fixed)	NA	NA	NA	NA	NA	NA	63.3274
SLC30A5	Pmin/Pmax observed, no tails	358.56 (250.63 – 527.95)	297.83 (244.79 – 337.22)	0.12	1	NA	NA	NA	NA	NA	NA	60.4083
Rag1	Pmin/Pmax fixed, right tails	424.55 (267.63 – 591.59)	341.84 (287.23 – 374.29)	0 (fixed)	1 (fixed)	NA	NA	169.01	0.14	NA	NA	53.77633

**Table 4:** Parameter estimates for the best fitting clines for 5 morphological traits using the package *HZAR*. For each trait, we present the best model, cline center (c), cline width (*w*), and AICc

Trait	Best Model	ω	c	AICc - 15-model best fit
Weight	Pmin/Pmax estimated, no tails	17.18 (0.98 – 307.37)	392.52 (314.01 – 440.56)	916.6954
Wing Chord	Pmin/Pmax estimated, no tails	53.82 (0.98 – 578.22)	390.811 (337.14 – 608.62)	1116.321
Bill Length	Pmin/Pmax estimated, no tails	353.06 (248.19 – 488.88)	292.73 (236.07 – 319.97)	227.5175
Plumage Amount	Pmin/Pmax estimated, no tails	450.36 (323.83 – 641.37)	284.05 (246.73 – 331.35)	1380.459
Plumage Definition	Pmin/Pmax estimated, no tails	283.15 (244.47 – 356.67)	328.57 (310.13 – 344.55)	1435.932

**Fig. 6:**
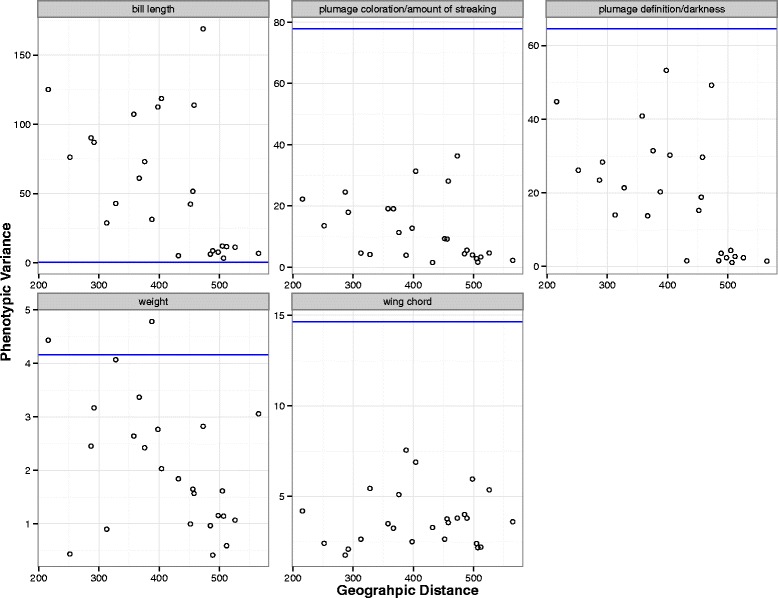
Variance for five morphological traits plotted for 22 sympatric *nelsoni* and *caudacutus* marshes along the sampling transect (254 individuals): bill length (*top left*), plumage coloration (*top middle*), plumage definition (*top right*), weight (*bottom left*), and wing chord (*bottom middle*). *V*_*max,*_ calculated as the maximum variance expected under a scenario of reproductive isolation, is plotted as a blue line. Traits with variance closer to *V*_*max*_ have reduced introgression

Estimates for cline width were narrower for traits related to the darkness and definition of plumage (349 km) compared to traits related to the amount of plumage streaking (380 km; Table 4). Estimates for cline center were similar (only 8 km difference in mean) between morphological traits and genetic markers. Phenotypic variance fluctuated across sympatric marshes. For the five morphological traits, we observed peaks in phenotypic variance that fell approximately between 350 and 450 km along the sampling transect, consistent with cline estimates for the center of the hybrid zone (Fig. 6). We observed peaks in phenotypic variance near the estimated center of the hybrid zone for weight and for traits related to definition/darkness of streaking; further, variance in weight exceeded *V*_*max*_ near the approximate zone center (Fig. 6). Variance in bill length in sympatric populations was greater than the variance observed in allopatric populations for all sampled marshes. The degree of introgression (ratio of *V*_*obs*_ and *V*_*max*_) was higher for wing chord (*V*_*obs*_*/V*_*max*_ = 0.5) and for plumage traits related to coloration and amount of streaking (*V*_*obs*_*/V*_*max*_ = 0.46) than it was for weight (*V*_*obs*_*/V*_*max*_ = 1.1) and plumage traits related to the definition and darkness of streaking (*V*_*obs*_*/V*_*max*_ = 0.82).

## Discussion

Species boundaries can remain distinct in the face of ongoing introgression, even if only a few regions of the genome remain differentiated while other regions become homogenized. Within the *caudacutus-nelsoni* hybrid zone, we found patterns indicative of strong selection (more abrupt slopes compared to a multi-locus average) for 5 out of 29 genetic markers despite extensive introgression in sympatric populations. We identified 42 % of the sampled individuals as admixed (hybrid index ranging from 0.1 to 0.9). The majority of these admixed individuals were backcrossed, with the very low proportion of recent generation hybrids in this system indicative of an advanced generation hybrid zone [42, 44]. The distribution of pure and admixed individuals appeared patchy across sympatric populations, with neighboring marshes exhibiting noticeable differences in genotypic compositions. Increased heterozygosity and *F*_*IS*_ at select marshes across the zone, including Weskeag and Chapman’s Landing (which are 112 and 128 km north and south from the center, respectively) support the idea that certain marshes facilitate mixing more than others.

The evolutionary history of *caudacutus* and *nelsoni* is complex; however, the leading hypothesis suggests that the current overlap zone is an area of secondary intergradation following a split during a Pleistocene glaciation event [51]. Consistent with this hypothesis, the results of this study provide evidence for secondary contact and contemporary introgression as opposed to incomplete lineage sorting. We found strong divergence across all markers in allopatric populations and high levels of admixture and a noticeable peak in phenotypic variation in sympatric populations. Greater genetic differentiation in allopatry (average *F*_*ST*_ between allopatric *caudacutus* and *nelsoni* = 0.313; locus-specific *F*_*ST*_ as high as 0.71) than in sympatry (average *F*_*ST*_ between sympatric *caudacutus* and *nelsoni* = 0.24; locus-specific *F*_*ST*_ as high as 0.61) suggests geographic structuring of alleles. Incomplete lineage sorting, alternatively, would manifest in random geographic distribution of ancestral alleles [55, 56]. Furthermore, the occurrence of recent generation hybrids in sympatric marshes, although in low frequency, points toward contemporary hybridization events between these species.

Estimates for cline width were highly variable among markers, ranging from 248 to 970 km, and were, on average, most narrow for mitochondrial and z-linked genes. Estimates for cline center, however, were consistent among marker types (genetic and morphological) falling around Yarmouth, Maine (328 km from locality 1). Previous field surveys identified *caudacutus* and *nelsoni* individuals co-occurring from Weskeag, Maine to Newburyport, Massachusetts (~208 km overlap zone; Hodgman et al., [39]). Consistent with the field estimates of the overlap zone, three of the markers analyzed in this study (ND2, SLC45A2, and *Ammo*006) exhibited cline widths in the 250 – 300 km range. The remaining markers had substantially larger cline widths, indicating extensive introgression and recombination within and well outside of the overlap zone.

Clines varying in width but constrained to the same center are indicative of differential introgression across the hybrid zone. This is consistent with predictions that hybrid zones act as a semi-permeable barrier for the exchange of genetic material between taxa [1]. We found differential introgression consistent with our a priori predictions for each marker type, including comparatively narrow cline estimates for mitochondrial, sex-linked, and select gene-associated, diagnostic markers relative to wide clines for neutral loci. This variable introgression across markers suggests that while most traits exhibit uninhibited movement, there are certain traits that do not freely cross the species’ boundaries and therefore may be important in reproductive isolation. The observed patterns can be explained by both selection against hybrids and adaptive divergence along a tidal marsh gradient as active mechanisms in shaping species boundaries between *caudacutus* and *nelsoni*.

Consistent with Haldane’s rule, we found that on average, mitochondrial and z-linked markers show reduced introgression compared to autosomal markers (including neutral and selected loci). Haldane’s rule predicts that fitness reductions should occur more often in hybrids of the heterogametic sex [25]; these differential fitness reductions appear to play an important role in speciation [57]. Reduced introgression of mitochondrial or sex-linked markers in organisms with ZW sex determination is an expectation of the dominance theory of the Dobzhansky-Muller incompatibility model [58–60]. This theory predicts that fitness reductions arise through the interaction of incompatible alleles, which evolved in allopatry. If these incompatible alleles are recessive, fitness reductions will be greater for the heterogametic sex if these genes are located on the sex chromosomes. In systems where females are the heterogametic sex, Haldane’s rule also predicts reduced introgression of mitochondrial markers because they are maternally inherited. There is extensive empirical support for Haldane’s rule [61], increasingly so in avian systems, including sterility (*Ficedula hypoleuca* and *F. albicollis*; [62]) and lower survival rates (*Larus argentatus* and *L. cachinnans*; [63] of hybrid females, and reductions in female-mediated gene flow (*Larus occidentalis* and *O. glaucescens*; [64] and *Aquila clanga* and *A. pomarina*; [65]). Accordingly, adaptive behavioral differences in pure *caudacutus* and *nelsoni* females associated with nesting synchrony in relation to tidal cycles [66] suggest a potentially important influence of differential fitness among pure and admixed females in shaping zone dynamics. Evidence for reduced survival in F1/F2 females provide further support for Haldane’s Rule in this system [67]. Nonetheless, other causes of the observed patterns of restricted introgression of mitochondrial and sex-linked genes cannot be discounted. Differences in marker-specific inheritance patterns, effective population sizes, genetic drift, and sex-biased dispersal can generate disparate rates of gene flow across the genome and lead to differential introgression across markers [1].

Only one marker (diagnostic microsatellite marker *Ammo006*) exhibited narrower clines than the z-linked and mitochondrial markers. Based on annotation with the zebra finch genome, *Ammo*006 was found to be associated with a gene that codes for a mitogen-activated protein kinase (MAPK; 52). Specifically, the MAPK superfamily consists of three distinct signaling pathways with roles linked to numerous cellular functions including immune responses, host-parasite interactions, and adaptive responses to thermal, osmotic, and oxygen stresses [68, 69]. Of particular interest is the response of MAPK to osmotic stress, which has been documented in a range of organisms [69], including in mammalian kidney [70] and liver astrocytes [71] and in the osmosensory signaling pathways of fish (*Fundulus heteroclitus*; [72]). MAPKs therefore may have a critical role in salinity adaptation [69] and may serve an important role in osmoregulatory functions of *A. caudacutus*. The transition from upland and brackish habitat (*nelsoni*) to salt marsh (*caudacutus*) presents major adaptive challenges to terrestrial vertebrates [45], and adaptive divergence across this salinity gradient may thus play an important role in reproductive isolation between the species [73]. Pathways related to osmotic stress (i.e., MAPK) would arguably be under strong selection in this system. The MAPK gene region likely plays an important ecological role for *A. caudacutus*, which exhibits a pre-Pleistocene association with tidal salt marshes [37, 47], and thereby a longer time to evolve adaptations to salt marshes compared to *A. nelsoni*, which exhibits a broader ecological niche, breeding also in grassland and brackish marshes and a more recent association with tidal marshes [38, 51, 74].

Restricted introgression of additional molecular and phenotypic traits provided evidence for selection on increased melanin in *A. caudacutus*, consistent with the hypothesized adaptive role for melanin in vertebrates that inhabit saltmarsh ecosystems [49]. Here we present two lines of support for this hypothesis. First, we found narrow cline estimates for the z-linked marker SLC45A2 (299 km) along with a more abrupt transition in slope compared to a multilocus average (+1.41). SLC45A2 (solute carrier family 45, member 2, protein) is associated with melanin-based pigmentation and has been linked to plumage phenotypes in birds, including silver and cinnamon colored phenotypes (*Gallus gallus* and *Coturnix japonica*; [75]) and the gray plumage of hooded crows (*Corvus cornix;* [76]). Similarly, mutations in SLC45A2 may relate to the differences in plumage coloration between *caudacutus* and *nelsoni. A. caudacutus* individuals have dark chestnut streaking patterns on the breast and flanks and dark chestnut backs, while *A. nelsoni* have gray streaking on the breast and flanks and more gray on the back [40, 42, 51]. Walsh et al. [42] found that plumage traits related to plumage darkness (particularly in the breast and flanks) are more strongly correlated with genotype. Secondly, we found that the introgression of traits related to plumage darkness was reduced (*V*_*obs*_*/V*_*max*_ = 0.82) compared to traits related to streaking amount (*V*_*obs*_*/V*_*max*_ = 0.46). Natural selection for the adaptive benefits of salt marsh melanism (including reduced predation risk and resistance to mechanical and bacterial degradation; [49, 77, 78]) may be reinforced by sexual selection [34]. The darkness of streaking in the breast and flanks may offer strong visual cues for individuals during mate selection.

We detected strong patterns of asymmetrical introgression across the 29 genetic markers, with 19 showing patterns of asymmetrical introgression toward *A. caudacutus* and 10 markers showing patterns of asymmetrical introgression toward *A. nelsoni*. A majority of these markers, including all of the markers that showed asymmetries toward *nelsoni*, displayed gradual slopes indicative of weak selection. Five markers exhibited abrupt clines and all of them showed patterns of asymmetry toward *caudacutus.* These findings are consistent with previous work suggesting that backcrossing is asymmetrical and biased toward *A. caudacutus* [40, 41], possibly due to differences in mating systems [40] or population density.

Both species exhibit an unusual mating system among emberizines, characterized by non-territoriality, lack of male parental care, and high levels of promiscuity facilitating intense male-male competition for receptive females [51, 79]. The two species differ in their mating tactics, however. *Nelsoni* males spend substantial time mate guarding and have more distinctive song and flight displays for attracting females [51, 66, 80]. *Caudacutus* males are highly polygamous and exhibit a scramble competition mating system whereby males search for and attempt to mate with multiple receptive females [37, 79]. Size differences between *nelsoni* and *caudacutus* males (14.9 – 19.2 g versus 19 – 24 g, respectively) may thus place *nelsoni* at a substantial competitive disadvantage when competing with *caudacutus* males to secure mates in sympatric marshes. Admixed females are thus more likely to backcross with *caudacutus* males leading to asymmetries. This is particularly true in sites toward the southern portion of the hybrid zone, where *caudacutus* males outnumber *nelsoni* males by approximately 4:1 [67]. Cline estimates for weight coupled with a peak in weight variance near the center of the zone provide supportive evidence that size is an important factor in shaping zone dynamics in this system. The cline for weight was the most abrupt of the five morphological traits analyzed, indicative of strong selection against intermediately sized individuals, which may be ineffective in securing mates using either of the mating tactics (direct male-male competition or flight displays and mate guarding). Furthermore, variance in weight at the center of the hybrid zone exceeded variance in allopatry, which may be indicative of character displacement with smaller *nelsoni* and larger *caudacutus* in sympatric versus allopatric populations.

Asymmetrical introgression has been documented in a number of avian contact zones [81–83] and may also be indicative of hybrid zone movement or of one species being displaced by the other. Moving hybrid zones leave tails of clines of unlinked neutral markers in their wake, giving the appearance of asymmetrical introgression [30]. Distinguishing hybrid zone expansion from asymmetrical introgression poses a challenge, and is best addressed with temporally replicated sampling. However, multiple alleles introgressing in one direction offers additional support for zone movement [30, 84]. Previous research has documented a potential southward expansion of *nelsoni* into the range of *caudacutus,* with *nelsoni* alleles documented as far south as Rhode Island [41]. Extensive field surveys also suggest a more pronounced decline in *caudacutus* abundance across their range in comparison to *nelsoni* (Correll et al., unpublished data); however, a direct temporal comparison of genetic data is required to test hypotheses of a hybrid zone expansion.

## Conclusions

In conclusion, we found support for hybrid zones acting as semi-permeable boundaries to foreign alleles across a tidal marsh gradient. While a majority of the markers used for this analysis showed patterns of weak selection and uninhibited movement across the hybrid zone, we found evidence for strong selection for a few molecular markers and plumage characteristics, consistent with evolutionary processes contributing to reproductive isolation. Specifically, we detected reduced introgression of mitochondrial and z-linked markers, providing evidence for Haldane’s rule, along with divergent selection for traits conferring adaptive benefits to tidal marshes. Despite the overall low genetic differentiation between *caudacutus* and *nelsoni*, niche differentiation may be driving ecological speciation between the species, with strong selective pressures for a few critical gene regions playing an important adaptive role. We conclude that adaptive divergence across a tidal marsh ecotone may promote isolating mechanisms and prevent the erosion of pure species boundaries in this system.

## Methods

### Geographic transect and sample collection

For this study, we used a previously published phenotypic and microsatellite genotypic dataset collected from the *caudacutus-nelsoni* hybrid zone [42], along with new DNA sequence data from two mitochondrial, 2 Z-lined, and one autosomal marker. The sampling design captured the extent of genetic and phenotypic variation across the hybrid zone, via 34 tidal marshes sampled along a linear, coastal transect from Lubec, Maine (44°49′22 N, 66°59′20 W) to Madison, Connecticut (41°15′46 N, 72°33′00 W; Fig. 1; Table 1) during the 2012 and 2013 breeding seasons (June – August). The marshes sampled for this study represent a gradient of tidal marsh habitat types (including coastal, bay, and upriver marshes) and exhibited a range of variation in salinity, vegetation structure, isolation, and tidal amplitude [48]. Within the hybrid zone, marshes were sampled approximately every 10 km; allopatric marshes were also sampled north and south of the hybrid zone (Fig. 1). We sampled 290 sparrows and collected a suite of morphological measurements from 254 of them [50]. Due to inadequate sample sizes (*n* = 2) in two of the sampled locations, we used data from 286 individuals from 32 sites for all analyses (Table 1; Fig. 1). Of these individuals, 37 sparrows were sampled from putatively pure *nelsoni* populations (*n* = 4 marshes), 52 individuals were sampled from putatively pure *caudacutus* populations (*n* = 6 marshes), and 197 individuals were sampled from sympatric populations (*n* = 22 marshes). Because this was the first extensive sampling and genetic evaluation of the hybrid zone, we initially defined all marshes outside of the currently hypothesized overlap zone of Hodgman et al. [39] to be putatively allopatric. We scored each individual sparrow for 13 plumage traits developed for evaluating levels of phenotypic introgression [40, 42]. Briefly, the plumage scores capture basic phenotypic differences between the species and include the color of the bill, the color and definition of the face and back, the width and definition of the whisker line and crown, and the amount and definition of the streaking on the back and flanks. We used digital calipers to measure tarsus length and bill length (nares to tip; mm), a wing-chord ruler to measure unflattened wing chord (mm), and a digital scale to measure weight (to the nearest 0.1 g). We collected blood samples (10 – 20 μl) from the brachial vein and transferred drops to Nobuto blood filter strips (Sterlitech, Kent, Washington) for storage at room temperature until later genetic analysis.

### Analysis of molecular markers

The genotypic dataset consisted of 24 microsatellite loci, including 12 putatively neutral, anonymous microsatellite loci [85–87] and 12 diagnostic microsatellites developed specifically to differentiate *caudacutus*, *nelsoni*, and hybrids [52]. Information on these markers and PCR amplification conditions can be found in Walsh et al. [42]. Owing to elevated divergence in the diagnostic marker panel relative to the anonymous microsatellites (*F*_*ST*_ = 0.4667 and *F*_*ST*_ = 0.15, respectively; [52]), we considered these two marker sets separately for some analyses and interpretation.

We also amplified each individual at two mitochondrial genes [1100 bp of NADH dehydrogenase subunit 2 (ND2); 356 bp of NADH dehydrogenase subunit 3 (ND3)], two z-linked genes [183 bp of solute carrier family 45, member 2 (SLC45A2), 724 bp of solute carrier family 30 (SLC30A5)], and one autosomal marker [900 bp of recombination activating gene 1 (RAG1); Primer information for these markers can be found in Additional file 6: Table S1]. PCR reactions included the following: 3 μl of eluted genomic DNA, 0.5 μM of each primer, 2.0 mM MgCl_2_, 1X PCR buffer (Promega, Madison, Wisconsin), 0.12 mM of deoxyribonucleotides, and 1 unit of Taq DNA polymerase (Promega). Cycling conditions were as follows: 35 – 40 cycles of 94 °C for 30 s, 46–60 °C for 45 s, 72 °C for 90 s, and a final extension step at 72 °C for 5 min. For the two shorter fragments (ND3, SLC45A2), we sequenced all individuals sampled along the geographic transect; sequences were visually inspected in 4Peaks (Nucleobytes, Amsterdam, NL) and aligned in Geneious Pro 4.7.6 (Biomatters Ltd, Auckland, NZ). We assigned sequences to one of two haplotypes (*caudacutus* or *nelsoni*) based on visual inspection of species-specific polymorphic sites identified in putatively allopatric populations. For the three longer fragments (ND2, SLC30A5, and RAG1), we sequenced a subset of 14 putatively pure individuals (based on morphology and microsatellite data) and designed a Restriction Fragment Length Polymorphism (RFLP) analysis to identify species-specific haplotypes in the PCR-amplified fragments. We digested amplified products in 25 μl reactions, with 10 μl template DNA, 0.2 μl of enzyme TseI, PstI, and MwoI (for ND2, SLC30A-5, and RAG-1 fragments respectively), and 2.5 μl of NEBuffer (New England BioLabs, Ipswich, MA, USA) and incubated according to manufacturer protocols. We resolved the resulting fragments on a 2 % agarose gel and assigned haplotypes (see Additional file 7: Table S2 for protocols and fragment patterns). For SLC30A-5 and RAG-1, the RFLP method allowed us to assign individuals to one of three haplotypes (*caudacutus*, *nelsoni*, or hybrid) based on the combination of observed banding patterns. We checked the validity of the RFLP assay using the 20 sequenced individuals (see above) and found no discrepancy between RFLP and sequence haplotypes.

### Population structure

For each site, we characterized genetic diversity using standard population genetic metrics. Specifically, we calculated unbiased estimates of expected and observed heterozygosities and tested for deviations from Hardy-Weinberg equilibrium in genepop V4 [88]. We also calculated genetic diversity metrics, including *F*_*IS*_, number of alleles, and allelic richness in fstat [89]. To quantify patterns of admixture for each site, we estimated a hybrid index (proportion of *caudacutus* alleles in an individual) and interspecific heterozygosity (proportion of an individuals’ genome with alleles inherited from both parental populations) for all individuals using the R package *introgress* [90, 91]. To identify markers under selection, we performed selection tests for all loci using an *F*_*ST*_ outlier approach [92] in the program lositan [93]. To test for genetic differentiation among populations, we calculated pairwise *F*_*ST*_ values and performed significance testing using 1000 permutations in fstat. To characterize genetic structure of the *caudacutus-nelsoni* hybrid zone, we used the Bayesian clustering approach of structure, version 2.3.2 [94]. structure uses membership proportions to assign all individuals into appropriate population clusters (*K*). We conducted five runs for each value of *K* = 1–5; each run consisted of a 300,000 burn-in followed by 200,000 iterations. We also ran structure with the 12 anonymous microsatellites separately to ensure that the gene-associated diagnostic markers were not biasing the results. Because we detected the same patterns with both marker sets, we ran all subsequent analyses with the full set of 24 microsatellites. We used the admixture model and assumed correlated allele frequencies [95]. We determined the most likely number of population clusters (*K*) using the Δ*K* method of Evanno et al. [96]; structure output was visualized using the program structure harvester [97]. Lastly, we tested for linkage disequilibrium using genepop.*P-*values for multiple comparisons were adjusted with the Bonferroni correction.

### Patterns of differential introgression: genomic clines

We used the R-package *introgress* to estimate genomic clines for each locus using a multinomial regression to estimate individual clines for each locus along an admixture gradient (represented by the hybrid index, calculated as the proportion of *caudacutus* alleles in an individual in *introgress*). Calculating hybrid index requires a priori definition of pure individuals of each parental species. In doing so, we took a conservative approach to minimize the potential for including introgressed individuals in our parental samples; we defined pure individuals as those sampled from allopatric populations >115 km north and south of the currently recognized overlap zone (Fig. 1; [42]). To identify loci that displayed deviations from neutral expectations, we compared the likelihoods of the regression models to a null model of neutral introgression. Null models were generated using parametric simulations described in Gompert & Buerkle [90]. Using this approach, a large simulated admixed population is generated based on expected genotype frequency distributions (estimated using hybrid index and heterozygosity values equal to the observed data). We simulated 2000 admixed individuals and adjusted all significance thresholds using the false discovery rate procedure [98]. Deviations from neutrality were summarized as either gradual clines (homozygote excess or deficit and/or heterozygote excess) or abrupt clines (heterozygote deficit indicative of disruptive selection or assortative mating; 90).

To test for concordance among genomic clines, we compared genomic clines at individual loci against the multilocus expectation using the logit-logistic model of Fitzpatrick [99]. This approach compares the mean hybrid index over all loci to a hybrid index for a focal locus. The logit-logistic model estimates two parameters: *u* gives the relative difference in cline position (positive values indicate a shift of the cline toward *caudacutus* and negative values indicate a shift toward *nelsoni*) and *v* gives the relative difference in slope (values greater than one indicate a more abrupt slope and stronger selection whereas values less than one indicate a more gradual slope and weaker selection compared to the multilocus average). Perfect concordance between a focal locus and the mean hybrid index would result in *u* = 0 and *v* =1. Thus, the expectation for equal introgression over all loci lies on the diagonal. We fit the parameters *u* and *v* using the function “mle2” in the R package *bblme*.

### Patterns of differential introgression: geographic clines

To evaluate the distribution of *caudacutus* and *nelsoni* alleles across the transect, we used the Metropolis-Hastings Markov chain Monte Carlo algorithm employed in the R package *HZAR* [100, 101] to fit a series of geographic cline models to allele frequencies for each genetic marker and a suite of morphological traits. We reduced the variation observed in the 24-microsatellite loci to a two-allele system using species-specific compound alleles [29, 102, 103]. Using this approach, each allele was assigned to a species group based on its coordinates on the first axis of a multiple correspondence analysis (MCA), conducted using the ca package in R. We ran fifteen separate models for each genetic marker, all of which estimated cline center (distance from sampling location 1, *c*) and width (1/maximum slope, *w*). The tested models included all possible combinations for fitting tails (none fitted, left only, right only, mirror tails, or both tails estimated separately) and for estimating allele frequencies at the cline ends (pMin, pMax; fixed to 0 and 1, observed values, or estimated values).

To compare introgression patterns between genetic and phenotypic data, geographic clines were also fitted to five morphological traits, including: bill length, wing chord, weight, and two separate groups of plumage traits. We used plumage traits predominantly related to 1) the amount of streaking and the width of plumage features observed on an individual (including crown width, malar width, face definition, streaking amount on the breast, flanks, and back, and color of the back) and 2) traits predominantly related to the darkness and definition of plumage traits on an individual (including bill darkness, face color, and definition of plumage on the crown, malar, breast, and flanks). Traits related to plumage streaking amount and those related to plumage darkness and definition were previously found to differ with respect to their correlation with genotype [42]. We ran five separate models for each morphological trait, all of which estimated trait mean and variance (right, left, and center) along with cline center and width; models varied in how the tails were fitted. We compared all models using Akaike information criterion corrected for small sample sizes (AICc) and considered models with the lowest AICc score as the best-fitting model [104].

### Patterns of differential introgression: phenotypic variance

In addition to comparing cline width and center for phenotypic traits, we quantified the variation in introgression among the morphometric and plumage features. We compared the phenotypic variance observed within our populations (*V*_*obs*_) to the maximum phenotypic variance expected under a hypothesis of complete reproductive isolation (*V*_*max*_) following the methods of Barton & Gale [105] and Gay et al. [29]. For each of our morphological traits, we compared *V*_*max*_ with *V*_*obs*_ (average variance calculated from the phenotypic clines) and measured the degree of introgression for each trait by the *V*_*obs*_*/V*_*max*_ ratio [29]. Traits involved in reproductive isolation are predicted to exhibit large variance in the center of the hybrid zone. The closer the observed peak in phenotypic variance (*V*_*obs*_) is to the variance expected under complete reproductive isolation (*V*_*max*_), the lower the degree of introgression is for that particular morphological trait.

## Availability of supporting data

The data sets supporting the results of this article are available in the Dryad repository: http://doi:10.5061/dryad.d433j. Additional data supporting the results of this article are included in additional files.

## Additional files

Additional file 1: Figure S1. Selection tests for 24 genetic markers for *A. caudacutus* and *A. nelsoni* populations. *F*_*ST*_ is plotted as a function of heterozygosity. Markers located in the gray area are within neutral expectation, markers in the red area are candidates for positive selection, and markers in the yellow section are candidates for balancing selection. (PDF 305 kb) Additional file 2: Figure S2. Determination of the number of genetic clusters (K) for *nelsoni* and *caudacutus* individuals sampled from 32 marshes using the ΔK (left) and LnPD (right) methods. (PDF 64 kb) Additional file 3: Figure S3. Heat map showing genetic differentiation (*F*_*ST*_) among the 32 sampled marshes. Populations range from Lubec, Maine (point 1) to Madison, Connecticut (point 32). The largest *F*_*ST*_ values are in red and the smallest values are in blue. (PDF 51 kb) Additional file 4: Figure S4. Plot of introgression patterns for 29 markers (24 microsatellites, 1 nuclear gene, 2 mitochondrial genes, and 2 z-linked genes). Each column represents a marker and each row represents an individual (allopatric individuals are not included). Colors correspond to parental alleles: 2 *nelsoni* alleles (white), 2 *caudacutus* alleles (black), and 1 allele from each parental population (grey). (PDF 74 kb) Additional file 5: Figure S5. Genomic clines estimated in *introgress* for *A. caudacutus* and *A. nelsoni.* Each plot represents a marker and includes the name of each locus and a P value from the analysis. Solid color lines represent genomic clines for *caudacutus* homozygote genotypes, dashed lines represent genomic clines for heterozygote genotypes. Shaded areas represent 95 % confidence intervals. (PDF 1572 kb) Additional file 6: Table S1. Primer information for the markers used in this study. Table includes locus name, annealing temperatures, fragment length, the number of individuals with haplotype data (either from sequencing or restriction fragment length polymorphism assays), primer sequences, and original reference for each primer. (PDF 67 kb) Additional file 7: Table S2. Restriction Fragment Length Polymorphism (RFLP) assay information for ND2, SLC30A-5, and RAG-1. Table includes the restriction enzyme used for each assay and the approximate sizes of bands produced for each species. Conditions for the restriction digests followed manufacturer protocols. (PDF 71 kb)
