# Supplementary material for: Differential introgression and the maintenance of species boundaries in an advanced generation avian hybrid zone
Source: BMC Evol Biol. 2016 Mar 22;16:65. doi: 10.1186/s12862-016-0635-y (PMC4802838; doi:10.1186/s12862-016-0635-y)

Additional File 1: Figure S1: Selection tests for 24 genetic markers for *A. caudacutus* and *A. nelsoni* populations. F_{ST} is plotted as a function of heterozygosity. Markers located in the gray area are within neutral expectation, markers in the red area are candidates for positive selection, and markers in the yellow section are candidates for balancing selection.

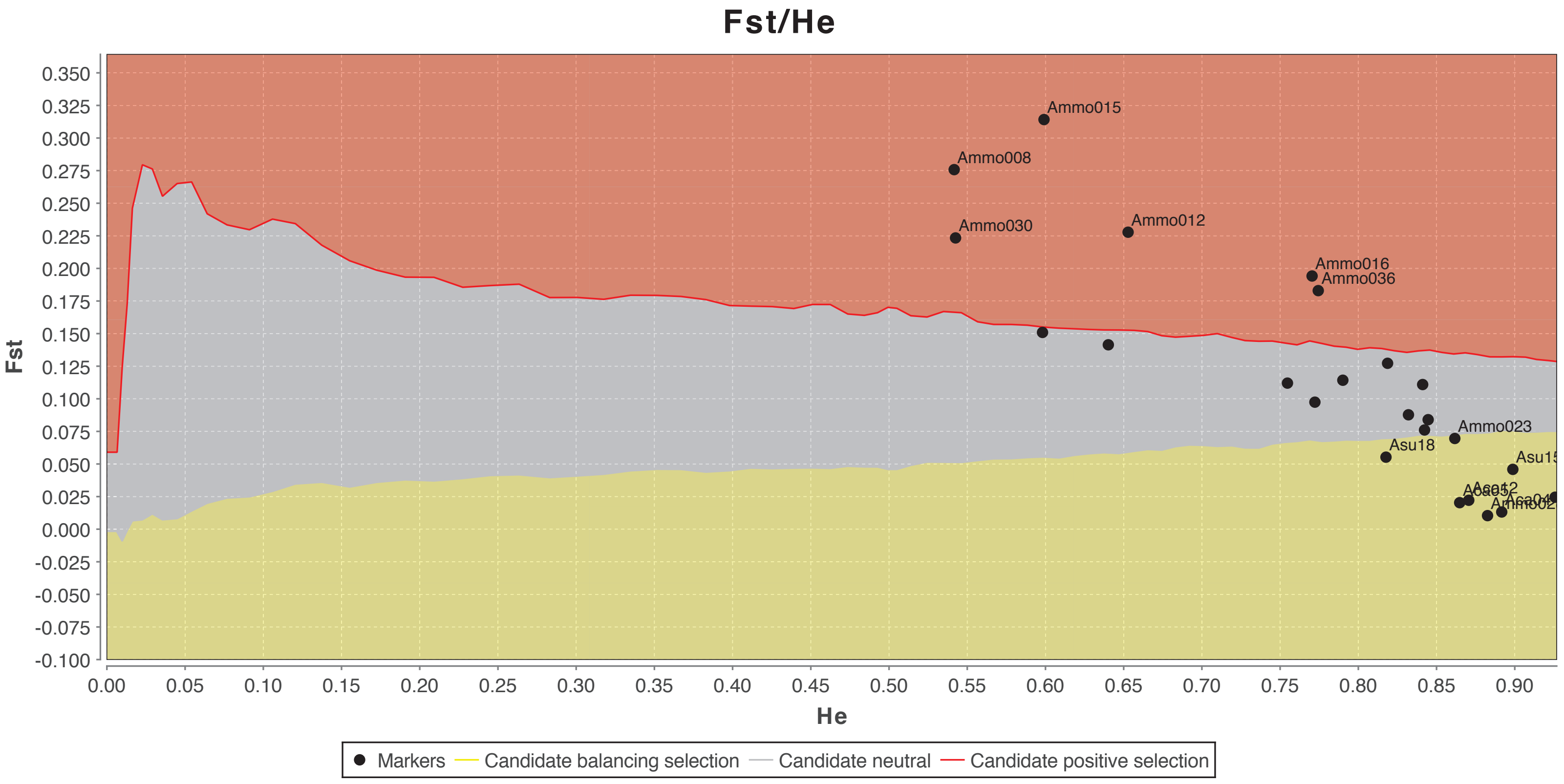

Supplement: Additional file 1: Figure S1. — Selection tests for 24 genetic markers for A. caudacutus and A. nelsoni populations. F ST is plotted as a function of heterozygosity. Markers located in the gray area are within neutral expectation, markers in the red area are candidates for positive selection, and markers in the yellow section are candidates for balancing selection. (PDF 305 kb) [file 12862_2016_635_MOESM1_ESM.pdf]
