# Supplementary material for: Differential introgression and the maintenance of species boundaries in an advanced generation avian hybrid zone
Source: BMC Evol Biol. 2016 Mar 22;16:65. doi: 10.1186/s12862-016-0635-y (PMC4802838; doi:10.1186/s12862-016-0635-y)

Additional File 2: Figure S2: Determination of the number of genetic clusters (K) for *nelsoni* and *caudacutus* individuals sampled from 32 marshes using the ΔK (left) and LnPD (right) methods.

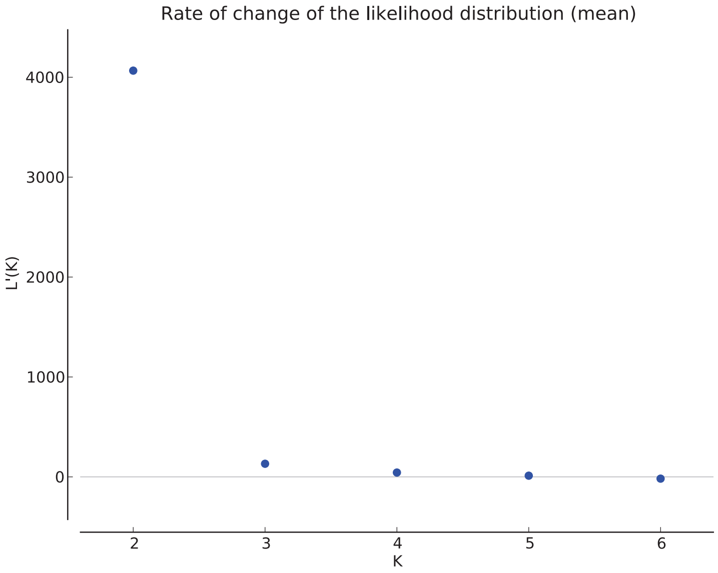

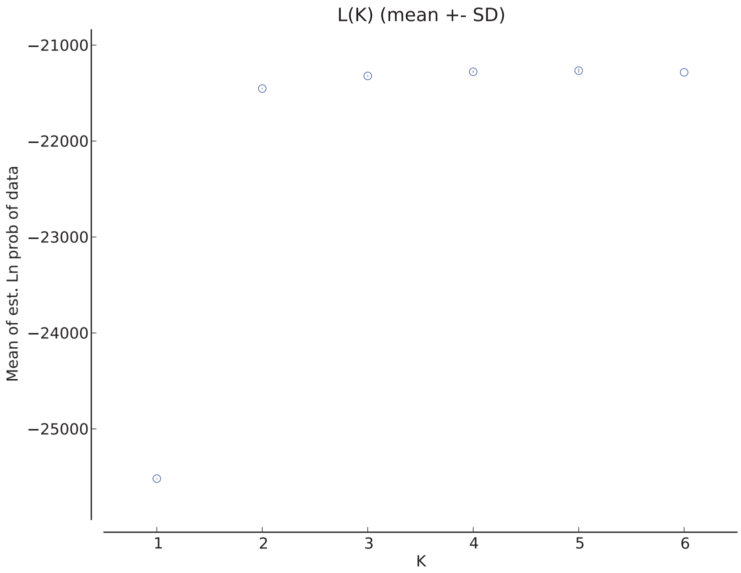

Supplement: Additional file 2: Figure S2. — Determination of the number of genetic clusters (K) for nelsoni and caudacutus individuals sampled from 32 marshes using the ΔK (left) and LnPD (right) methods. (PDF 64 kb) [file 12862_2016_635_MOESM2_ESM.pdf]
