# Supplementary material for: Differential introgression and the maintenance of species boundaries in an advanced generation avian hybrid zone
Source: BMC Evol Biol. 2016 Mar 22;16:65. doi: 10.1186/s12862-016-0635-y (PMC4802838; doi:10.1186/s12862-016-0635-y)

Additional File 3: Figure S3: Heat map showing genetic differentiation (F_{ST}) among the 32 sampled marshes. Populations range from Lubec, Maine (point 1) to Madison, Connecticut (point 32). The largest F_{ST} values are in red and the smallest values are in blue.

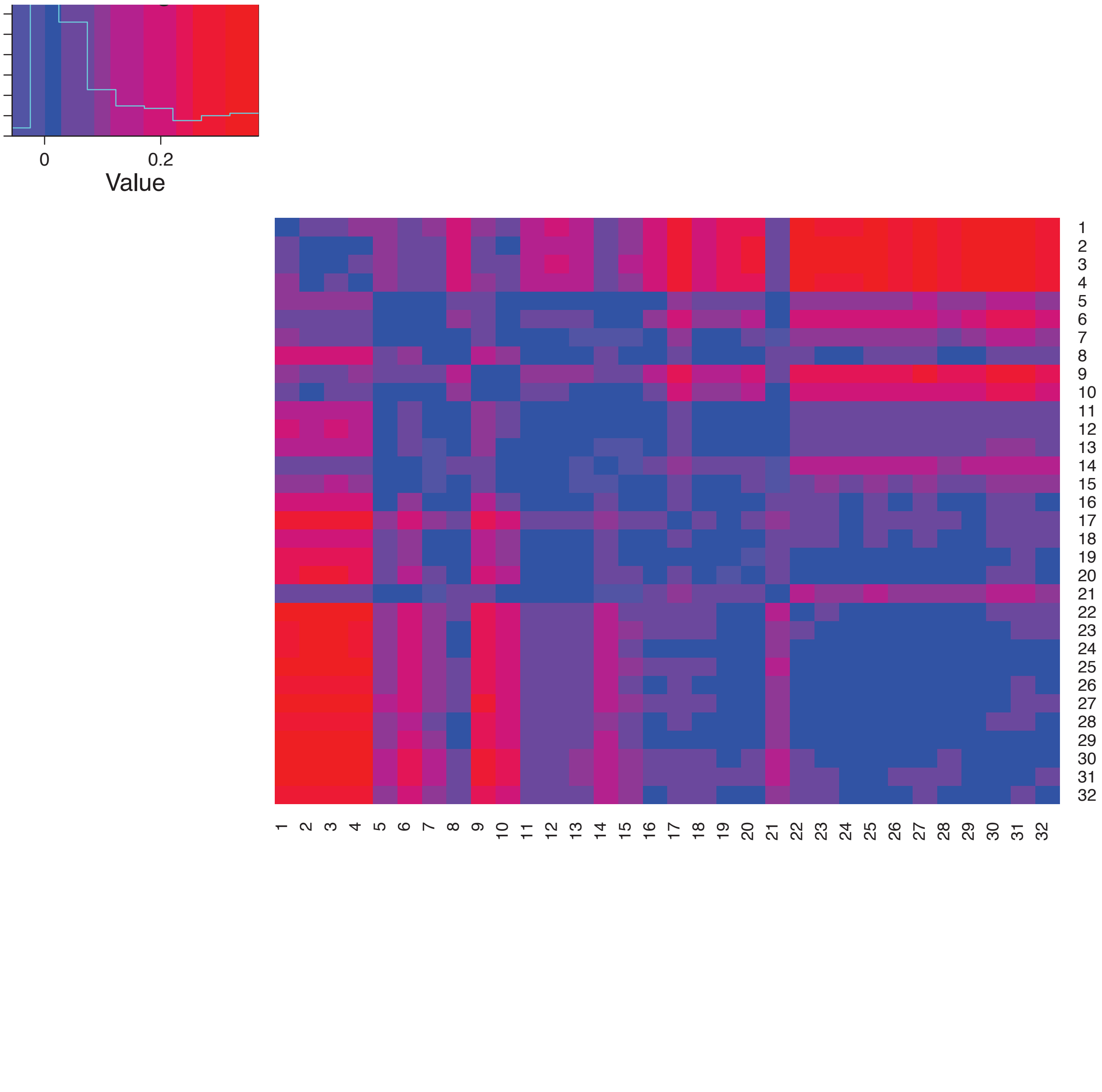

Supplement: Additional file 3: Figure S3. — Heat map showing genetic differentiation (F ST) among the 32 sampled marshes. Populations range from Lubec, Maine (point 1) to Madison, Connecticut (point 32). The largest F ST values are in red and the smallest values are in blue. (PDF 51 kb) [file 12862_2016_635_MOESM3_ESM.pdf]
