# Supplementary material for: Differential introgression and the maintenance of species boundaries in an advanced generation avian hybrid zone
Source: BMC Evol Biol. 2016 Mar 22;16:65. doi: 10.1186/s12862-016-0635-y (PMC4802838; doi:10.1186/s12862-016-0635-y)

Additional File 4: Figure S4: Plot of introgression patterns for 29 markers (24 microsatellites, 1 nuclear gene, 2 mitochondrial genes, and 2 z-linked genes). Each column represents a marker and each row represents an individual (allopatric individuals are not included). Colors correspond to parental alleles: 2 *nelsoni* alleles (white), 2 *caudacutus* alleles (black), and 1 allele from each parental population (grey).

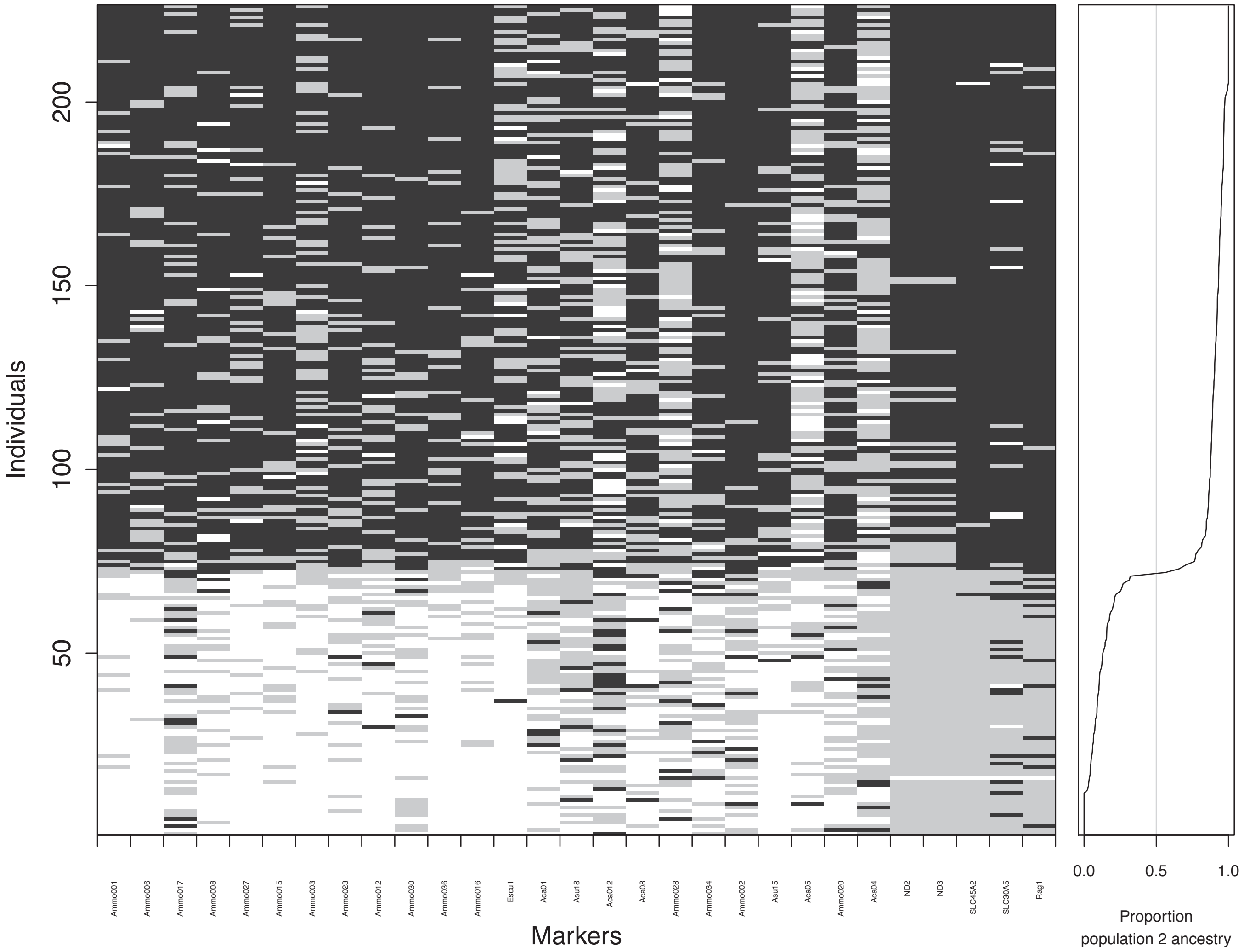

Supplement: Additional file 4: Figure S4. — Plot of introgression patterns for 29 markers (24 microsatellites, 1 nuclear gene, 2 mitochondrial genes, and 2 z-linked genes). Each column represents a marker and each row represents an individual (allopatric individuals are not included). Colors correspond to parental alleles: 2 nelsoni alleles (white), 2 caudacutus alleles (black), and 1 allele from each parental population (grey). (PDF 74 kb) [file 12862_2016_635_MOESM4_ESM.pdf]
