# Supplementary material for: Differential introgression and the maintenance of species boundaries in an advanced generation avian hybrid zone
Source: BMC Evol Biol. 2016 Mar 22;16:65. doi: 10.1186/s12862-016-0635-y (PMC4802838; doi:10.1186/s12862-016-0635-y)

Additional File 5: Figure S5: Genomic clines estimated in *introgress* for *A. caudacutus* and *A. nelsoni.* Each plot represents a marker and includes the name of each locus and a P value from the analysis. Solid color lines represent genomic clines for *caudacutus* homozygote genotypes, dashed lines represent genomic clines for heterozygote genotypes. Shaded areas represent 95% confidence intervals.

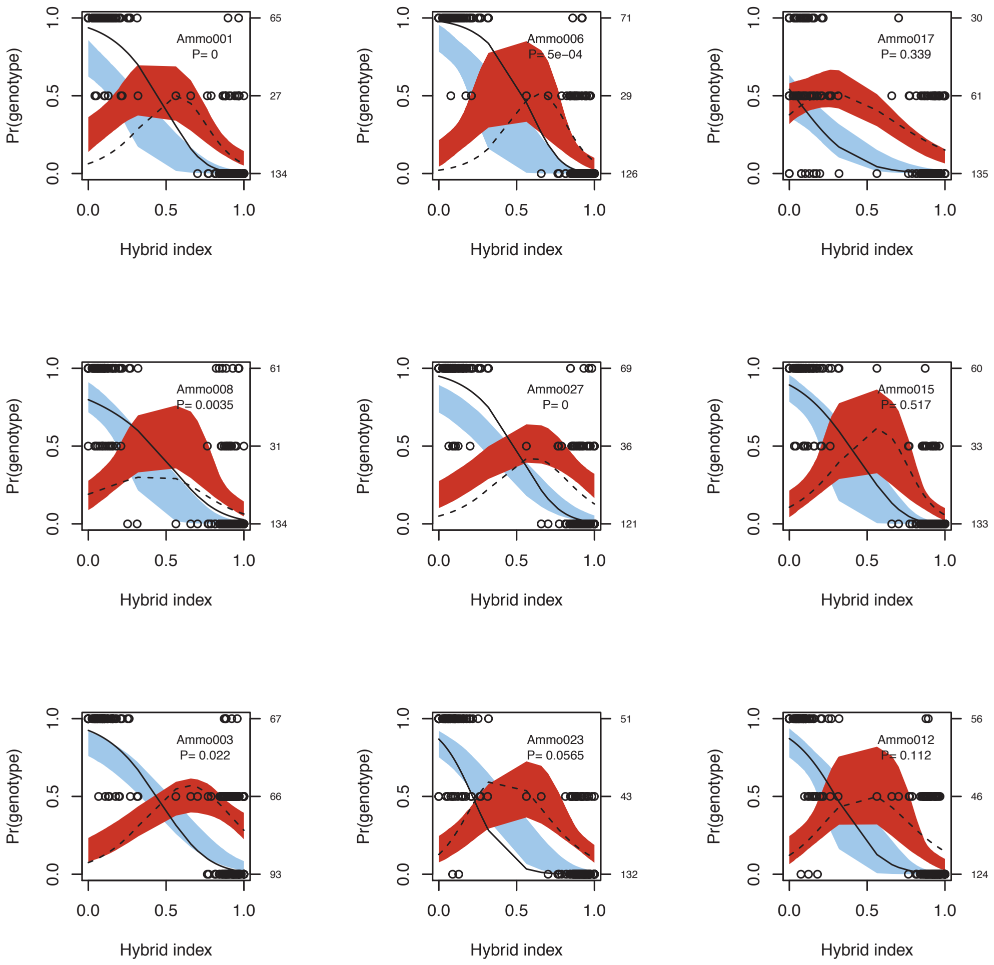

Additional File 5: Figure S5: *Continued*

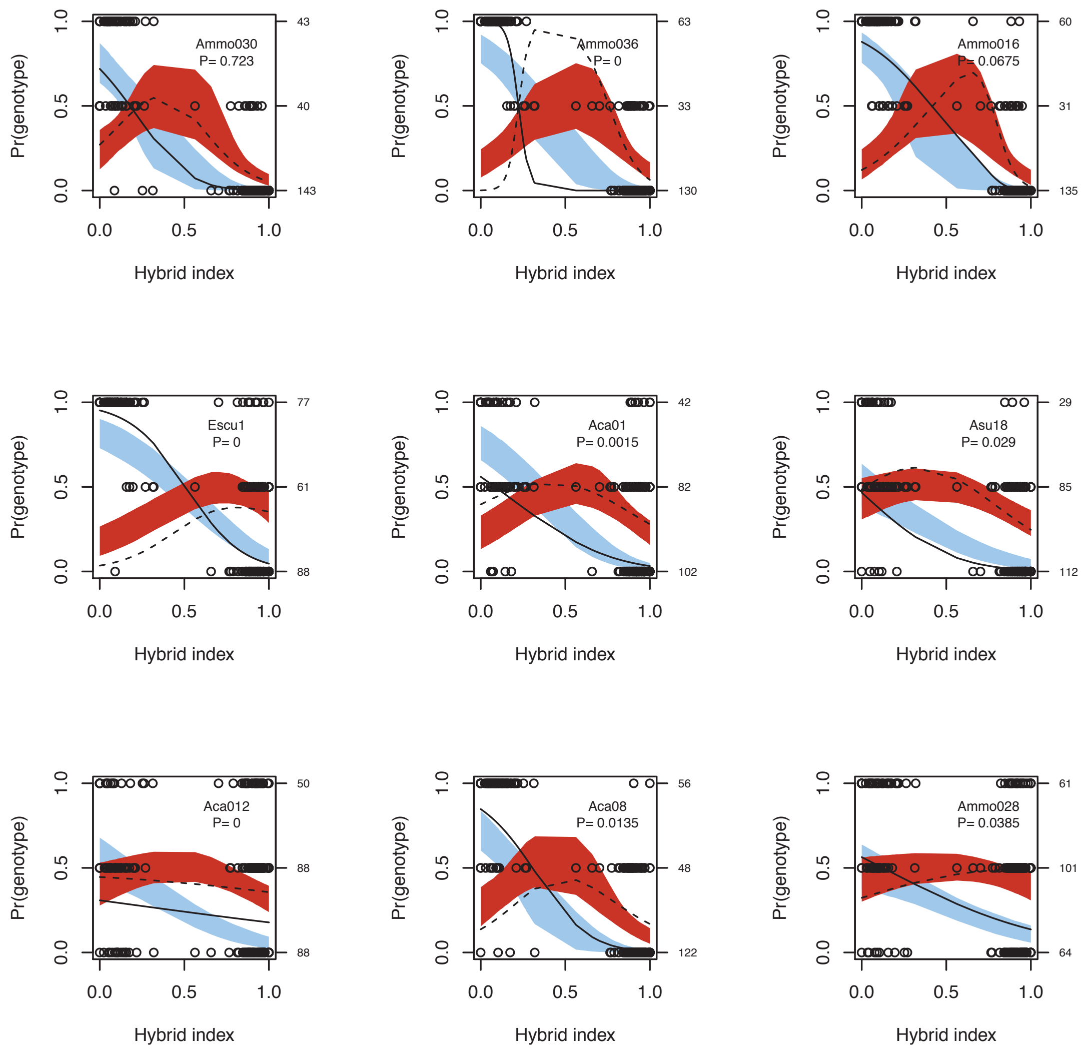

Additional File 5: Figure S5: *Continued*

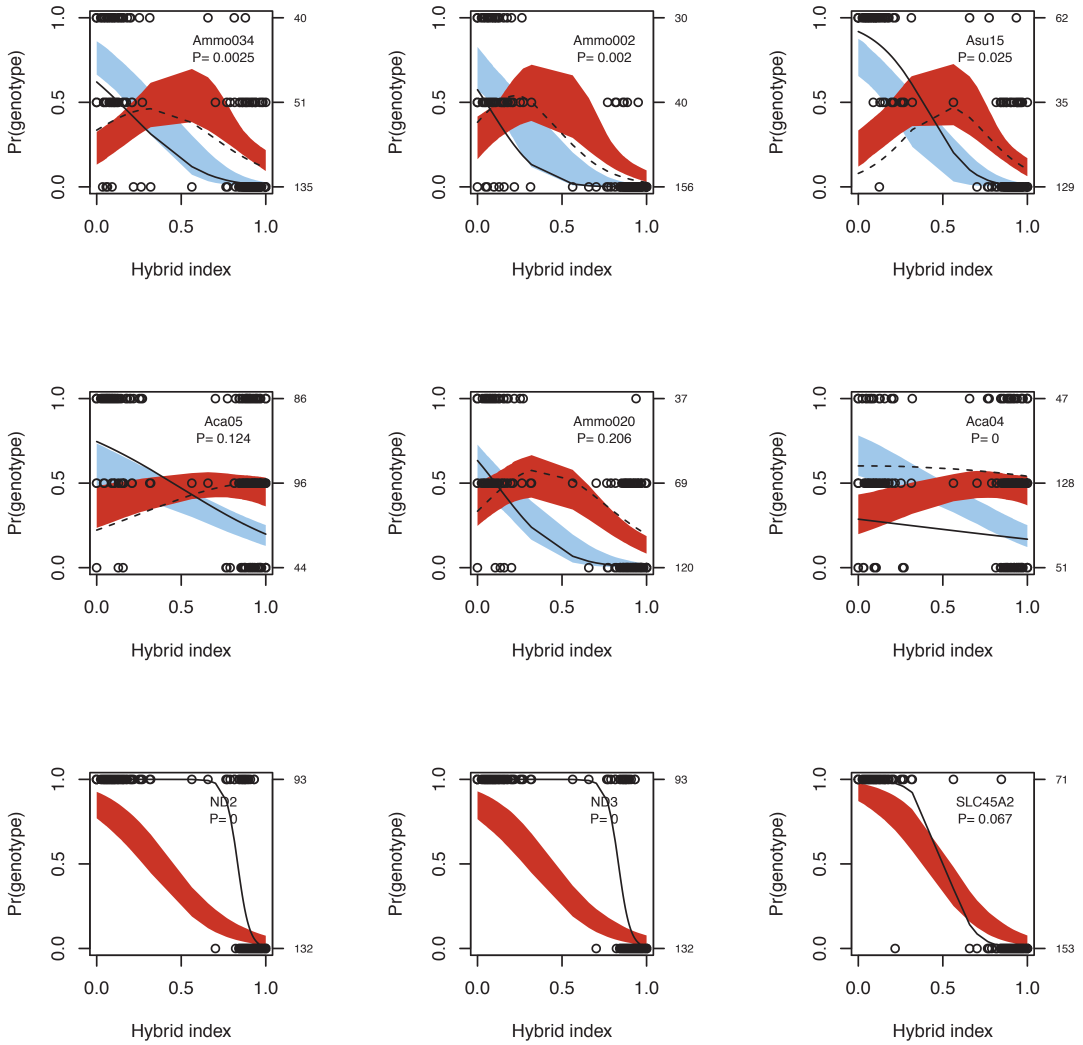

Additional File 5: Figure S5: *Continued*

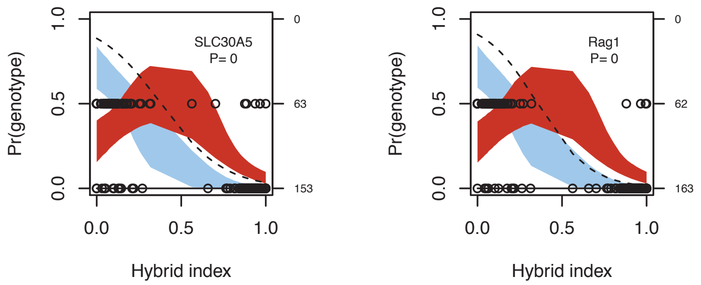

Supplement: Additional file 5: Figure S5. — Genomic clines estimated in introgress for A. caudacutus and A. nelsoni. Each plot represents a marker and includes the name of each locus and a P value from the analysis. Solid color lines represent genomic clines for caudacutus homozygote genotypes, dashed lines represent genomic clines for heterozygote genotypes. Shaded areas represent 95 % confidence intervals. (PDF 1572 kb) [file 12862_2016_635_MOESM5_ESM.pdf]
