# Supplementary material for: Differential introgression and the maintenance of species boundaries in an advanced generation avian hybrid zone
Source: BMC Evol Biol. 2016 Mar 22;16:65. doi: 10.1186/s12862-016-0635-y (PMC4802838; doi:10.1186/s12862-016-0635-y)
Supplement: Additional file 6: Table S1. — Primer information for the markers used in this study. Table includes locus name, annealing temperatures, fragment length, the number of individuals with haplotype data (either from sequencing or restriction fragment length polymorphism assays), primer sequences, and original reference for each primer. (PDF 67 kb) [file 12862_2016_635_MOESM6_ESM.pdf]

Additional File 6: Table S1:Primer information for the markers used in this study. Table includes locus name, annealing temperatures, fragment length, the number of individuals with haplotype data (either from sequencing or restriction fragment length polymorphism assays), primer sequences, and original reference for each primer

Locus	Annealing Temperature C°	Length (bp)	N	Primer Sequence	Reference
ND3 (mitochondrial)	46°	356	284	F: GACTTCCAATCTTTAAAATCTGG<br>R: GATTTGTTGAGCCGAAATCAAC	Chesser 1999
ND2 (mitochondrial)	58°	1100	284	F: GGCCCATACCCCGRAAATG<br>R: ACTCTTRTTTAAGGCTTTGAAGGC	Sorenson et al. 1999
SLC45A-2 (Z-linked)	60°	183	283	F: TCAACATTTAGGCTGCATTCC<br>R: ACAGGGGACTGTATCCTTGC	Made for this study
SLC30A-5 (Z-linked)	59°	724	271	F: TGTTAATGCCAACATTTCTTCA<br>R: ATTGCCCCGGTTTATTAAGG	Made for this study
RAG-1 (nuclear)	60°	595	284	F: GGCCAGTGGATGATGAAACT<br>R: TAGCTGCCCATAACCCTGAT	Made for this study
