# Supplementary material for: Differential introgression and the maintenance of species boundaries in an advanced generation avian hybrid zone
Source: BMC Evol Biol. 2016 Mar 22;16:65. doi: 10.1186/s12862-016-0635-y (PMC4802838; doi:10.1186/s12862-016-0635-y)
Supplement: Additional file 7: Table S2. — Restriction Fragment Length Polymorphism (RFLP) assay information for ND2, SLC30A-5, and RAG-1. Table includes the restriction enzyme used for each assay and the approximate sizes of bands produced for each species. Conditions for the restriction digests followed manufacturer protocols. (PDF 71 kb) [file 12862_2016_635_MOESM7_ESM.pdf]

Additional File 7: Table S2:Restriction Fragment Length Polymorphism (RFLP) assay information for ND2, SLC30A-5, and RAG-1. Table includes the restriction enzyme used for each assay and the approximate sizes of bands produced for each species. Conditions for the restriction digests followed manufacturer protocols.

Marker	Restriction Enzyme	Banding Patterns for *nelsoni*	Banding Patterns for *caudacutus*
ND2	TseI	832 bp, 206 bp	669 bp, 206 bp, 163 bp
SLC30A-5	PstI	724 bp	324 bp, 400 bp
RAG-1	MwoI	595 bp	316 bp, 279 bp
